# Recent changes in human resources for health and health facilities at the district level in Indonesia: evidence from 3 districts in Java

**DOI:** 10.1186/1478-4491-9-5

**Published:** 2011-02-13

**Authors:** Peter Heywood, Nida P Harahap, Siska Aryani

**Affiliations:** 1 Menzies Centre for Health Policy, University of Sydney, NSW, Australia; 2 Jalan Bukit Dago Selatan, Bandung, West Java Province, Indonesia; 3 Lecturer, Politeknik Kesehatan, Bandung, West Java Province, Indonesia

## Abstract

**Background:**

There is continuing discussion in Indonesia about the need for improved information on human resources for health at the district level where programs are actually delivered. This is particularly the case after a central government decision to offer doctors, nurses and midwives on contract the chance to convert to permanent civil service status. Our objective here is to report changes between 2006 and 2008 in numbers and employment status of health staff in three districts following the central government decision.

**Methods:**

Information was derived from records at the district health office and, where necessary for clarification, discussions with district officials.

**Results:**

Across the three districts and all public sector provider categories there was an increase of almost 680 providers between 2006 and 2008 - more than 300 nurses, more than 300 midwives and 25 doctors. The increases for permanent public servants were proportionately much greater (43%) than the total (16%). The increase in those who are permanent civil servants was greatest for nurses (51%) and midwives (35%) with corresponding decreases in the proportion of staff on contract. There was considerable variation between the three districts.

**Conclusions:**

There has been a significant increase in the number of healthcare providers in the 3 districts surveyed and the proportion now permanent public servants has increased even more than the increase in total numbers. The changes have the effect of increasing the proportion of total public expenditure allocated to salaries and reducing the flexibility of the districts in managing their own budgets. Because public servants are allowed private practice outside office hours there has also been an increase in the number of private practice facilities offering health care. These changes illustrate the need for a much improved human resources information system and a coherent policy to guide actions on human resources for health at the national, provincial and district levels.

## Background

We earlier reported on human resources for health [1] and health facilities [2] at the district level in Indonesia in 2006. For that report we enumerated healthcare providers (doctors, nurses and midwives) and health facilities, both public and private, in 15 districts on Java.

In summary, for healthcare providers in 2006:

• Approximately half of all three professional groups (doctors, nurses and midwives) were permanent civil servants (PNS);

• Central government contracts (PTT) were of most importance for midwives and were least important for doctors;

• Local contracts^1 ^were most important for nurses (41% across the 15 districts);

• The private sector as primary source of employment was most important for doctors (37% across the 15 districts).

For facilities^2^:

• 86% of all facilities were solo-providers, and these were all private; part-time private practice by nurses was the largest group of solo-provider facilities, despite the fact that private practice by nurses is illegal;

• 13% of facilities were multiple providers giving both inpatient and outpatient care; and

• 1% of facilities were multiple-provider giving both inpatient and outpatient care - they are a mixture of both public and private.

Our earlier report on personnel [1] also pointed out that the civil service status of public sector employees was set to change from that enumerated in 2006 as the government had decided to offer those on contract (including both central and local contracts) who met a minimum set of criteria the opportunity to convert to permanent civil service status (PNS - Pegawai Nasional Sipil). At the same time a central government law prohibited districts from hiring health staff on local contracts or as volunteers [3,4] even though both of these categories had been important in allowing districts some flexibility in numbers and skills mix for their staff.

Further, there is an interaction between personnel policy and the situation with health facilities. Because public service doctors and midwives (both PNS and contract) have the right to private practice an increase in their number in a district is likely to result in an increase in the number of private part-time solo-provider facilities through which the public servant doctors and midwives offer services after hours. The same situation would apply with nurses as, although private practice by nurses is not legal, it is widely acknowledged that they do so as well. The increased numbers of health staff may also stimulate an increase in the number of multi-provider private facilities such as treatment clinics.

Our objective is to determine the extent and effect of any increase in the number of civil service doctors, nurses and midwives after the central government policy change. In order to do so we re-censused these groups and re-enumerated health facilities for the year 2008 in three of the five districts we had earlier surveyed in West Java Province (Ciamis District, Garut District, Sukabumi District) in 2006. Here we report the results and discuss the implications for policies for human resources for health and development of the sector.

## Methods

The work was carried out in mid-2009. The methods were the same as used earlier and are described in detail in [1] and [2]. The information was collected in each district where the primary source of data was the district health office and the district hospital. All health care providers who do not work for the government but have a private practice in which health care is provided should be licensed by the government; our list was supplemented by these sources as well. For each provider we recorded their employment status and primary place of work.

## Results

### Healthcare providers

The results are shown in Table 1. In summary, between 2006 and 2008 across the 3 districts:

**Table 1:** Healthcare staff in three districts of West Java Province by staff category and provider type, 2006 and 2008 (see Note 1 below)

Employment status	General doctor			Nurse	Midwife		All providers		Percent	
	2006	2008	2006	2008	2006	2008	2006	2008	2006	2008
**Ciamis District**										
Permanent public servant (PNS)	50	55	430	657	320	438	800	1150	57	74
Various forms of contract to public sector (see Note 2)	8	17	371	195	119	93	498	305	35	20
Private practice full-time	38	35	34	25	33	39	105	99	7	6
Total	96	107	835	877	472	570	1403	1554	100	100
										
**Garut District**										
Permanent public servant (PNS)	59	66	573	758	277	397	909	1221	57	63
Various forms of contract to public sector (see Note 2)	33	27	411	429	173	176	617	632	39	33
Private practice full-time	53	68	0	0	18	23	71	91	4	5
Total	145	161	984	1187	468	596	1597	1944	100	100
										
**Sukabumi District**										
Permanent public servant (PNS)	52	84	218	430	246	303	516	817	43	59
Various forms of contract to public sector (see Note 2)	55	21	350	221	123	170	528	412	44	30
Private practice full-time	99	99	20	21	37	33	156	153	13	11
Total	206	204	588	672	406	506	1200	1382	100	100
										
**Three districts**										
Permanent public servant (PNS)	161	205	1221	1845	843	1138	2225	3188	53	65
Various forms of contract to public sector (see Note 2)	96	65	1132	845	415	439	1643	1349	39	28
Private practice full-time	190	202	54	46	88	95	332	343	8	7
Total	447	472	2407	2736	1346	1672	4200	4880	100	100
2008 minus 2006		25		329		326		680		

Note 1: data for 2006 from Reference 1, Table 13a; data for 2008 from re-census in June 2009.

Note 2: 'Various forms of contract' includes central, district and facility contracts (PTT, kontrak), volunteers (sukwan) and daily hires (bidan harian lepas).

• there was an increase of 680 staff in the health sector, more than 300 nurses, more than 300 midwives and 25 doctors, an increase of 16%. There was considerable variation between districts with the increase being greatest in Garut District (347) and least in Ciamis District (151).

• the increase in numbers was smallest for doctors (6%) and greatest for midwives 24%;

The increases for permanent public servants (PNS) were proportionately much greater than the total

• across all districts the number of PNS (doctors, nurses and midwives) increased by 43%

• the increase was greatest for PNS nurses (51%) (with considerable variation between districts from 32% in Garut to 97% in Sukabumi); in 2006 49% of public sector nurses were PNS, the remainder were on local contracts as central contracts (PTT) have never been available to nurses. By 2008, more than 600 nurses, all on local contracts, had converted to PNS. At the same time, an additional 300 nurses were hired on local contracts so that the net change in the number of nurses was an increase of 329 (Table 1).

• The increase for PNS midwives was 35% overall (with variation from 23% in Sukabumi to 43% in Garut). In 2006, 67% of public sector midwives were PNS, one-fifth were on central contracts and less than one-sixth on local contracts. In 2008, almost 300 midwives, mostly on central contracts, were converted to PNS. An additional 300 were hired on contracts with the result that the net change in the number of midwives was in increase of 326.

• the increase was least for PNS doctors, 27% overall (with considerable variation between districts). In 2006, 63% of public sector doctors were PNS and more than two-thirds of the remainder were on central contracts. In 2008, 44 doctors, almost all on central contracts, were converted to PNS. At the same time the number of doctors in sole private practice increased by 12. The result was an increase in the number of doctors by 25.

• The increase across the 3 districts was greatest for nurses and midwives - an average of more than 100 per district for both midwives and nurses.

Across doctors, nurses and midwives, the proportion of PNS has increased from 53% in 2006 to 65% in 2008 and for contract staff has fallen from 39% to 28% over the same period. The reduction in contract staff was particularly marked for nurses who were all on local contracts. Nevertheless, it is important to note that all districts are still hiring considerable numbers of healthcare providers on various forms of local contracts, despite the ban on such hiring by the central government. In some cases the law is ignored, in others it is circumvented merely by using a different name for the local contract category. The overall result is that the proportion of health staff now PNS has increased and, as many of those converted to PNS have been replaced by additional local contract staff, the total number of health staff has increased.

There was a small increase in the number of healthcare staff in full-time private practice and the proportion of the total remained virtually unchanged between 7% and 8%.

### Health facilities

The results are summarized in Tables 2, 3 and 4 for Ciamis, Garut and Sukabumi Districts, respectively, and across the three districts in Table 5. Given that doctors and midwives have the right to private practice and nurses also set up private practices even though they are not allowed to do so under the regulations, the total number of private practice facilities would be expected to increase with any increase in the number of public sector staff. This was indeed the case - an increase of 511 facilities overall, 369 of which were solo-provider facilities and 142 multiple-provider facilities.

**Table 2:** Facilities in Ciamis District, West Java Province, 2006 and 2008.

Ciamis District	2006	2008
Public hospital (Rumah Sakit Umum Daerah (RSUD))	1	1
Private hospital (Rumah Sakit Swasta (RSUS))	3	3
Hospital for women and children (Rumah Sakit Ibu dan Anak (RSIA))	0	0
Women's hospital (Rumah Sakit Bersalin (RSB))	0	0
Maternity clinic (Rumah Bersalin (RB))	2	2
Health center (Pusat Kesehatan Masyarakat (Puskesmas))	51	51
Auxiliary health center (Puskesmas pembantu (Pustu))	82	118
Treatment clinic (Balai pengobatan (BP))	75	95
Sub-total (multiple-provider facility)	214	270
Village midwife (Bidan di desa (BDD)/Pondok Bersalin Desa (Polindes))	273	275
Doctor in full-time private practice	38	39
Doctor in part-time private practice	58	59
Nurse in part-time private practice	501	511
Midwife in full-time private practice	152	186
Sub-total (solo-provider facility)	1022	1070
Total	1236	1340

**Table 3:** Facilities in Garut District, West Java Province, 2006 and 2008

Garut District	2006	2008
Public hospital (Rumah Sakit Umum Daerah (RSUD))	1	1
Private hospital (Rumah Sakit Swasta (RSUS))	1	1
Hospital for women and children (Rumah Sakit Ibu dan Anak (RSIA))	0	0
Women's hospital (Rumah Sakit Bersalin (RSB))	0	0
Maternity clinic (Rumah Bersalin (RB))	2	6
Health center (Pusat Kesehatan Masyarakat (Puskesmas))	62	62
Auxiliary health center (Puskesmas pembantu (Pustu))	132	135
Treatment clinic (Balai pengobatan (BP))	8	69
Sub-total (multiple-provider facility)	206	274
Village midwife (Bidan di desa (BDD)/Pondok Bersalin Desa (Polindes))	305	370
Doctor in full-time private practice	53	68
Doctor in part-time private practice	92	87
Nurse in part-time private practice	590	712
Midwife in full-time private practice	18	23
Sub-total (solo-provider facility)	1058	1260
Total	1264	1534

**Table 4:** Facilities in Sukabumi District, West Java Province, 2006 and 2008.

Sukabumi District	2006	2008
Public hospital (Rumah Sakit Umum Daerah (RSUD))	3	3
Private hospital (Rumah Sakit Swasta (RSUS))	2	2
Hospital for women and children (Rumah Sakit Ibu dan Anak (RSIA))	0	0
Women's hospital (Rumah Sakit Bersalin (RSB))	0	0
Maternity clinic (Rumah Bersalin (RB))	11	11
Health center (Pusat Kesehatan Masyarakat (Puskesmas))	57	57
Auxiliary health center (Puskesmas pembantu (Pustu))	98	110
Treatment clinic (Balai pengobatan (BP))	47	53
Sub-total (multiple-provider facility)	218	236
Village midwife (Bidan di desa (BDD)/Pondok Bersalin Desa (Polindes))	283	351
Doctor in full-time private practice	99	99
Doctor in part-time private practice	118	135
Nurse in part-time private practice	353	391
Midwife in full-time private practice	37	33
Sub-total (solo-provider facility)	890	1009
Total	1108	1245

**Table 5:** Facilities in three districts (Ciamis, Garut, Sukabumi) combined, 2006 and 2008.

Three district combined	2006	2008
Public hospital (Rumah Sakit Umum Daerah (RSUD))	5	5
Private hospital (Rumah Sakit Swasta (RSUS))	6	6
Hospital for women and children (Rumah Sakit Ibu dan Anak (RSIA))	0	0
Women's hospital (Rumah Sakit Bersalin (RSB))	0	0
Maternity clinic (Rumah Bersalin (RB))	15	19
Health center (Pusat Kesehatan Masyarakat (Puskesmas))	170	170
Auxiliary health center (Puskesmas pembantu (Pustu))	312	363
Treatment clinic (Balai pengobatan (BP))	130	217
Sub-total (multiple-provider facility)	638	780
Village midwife (Bidan di desa (BDD)/Pondok Bersalin Desa (Polindes))	861	996
Doctor in full-time private practice	190	206
Doctor in part-time private practice	268	281
Nurse in part-time private practice	1444	1614
Midwife in full-time private practice	207	242
Sub-total (solo-provider facility)	2970	3339
Total	3608	4119

• For solo-provider facilities, more than half the increases are the practices of nurses, most of the remainder are village midwives.

• For multiple-provider facilities, the increase is basically shared between treatment centres and auxiliary health centres.

## Discussion

The decision by the government to convert contract staff to PNS had three main effects. First, it increased the total number of permanent civil servants in the health sector in these three districts by 43% - as a result the proportion of healthcare staff who are PNS increased from 53% to 65% while the proportion on contract decreased from 39% to 28%. Second, because the district governments then hired additional staff on local contracts, the total number of public sector healthcare providers increased by 16%. Third, there has been a significant increase in the public sector salary costs, partly due to the increase in total number of public sector staff (including the new hires on local contracts to, at least partially, replace those converted to PNS) and partly due to the increased commitments of the government to the benefits, including pensions, for the PNS staff.

There were considerable differences between the professions - overall the number of PNS nurses increased by 51%, midwives 35% and doctors 27%. There are also considerable differences between the districts.

These are very significant changes in the health sector within the space of a year. The results presented here for three districts, the outcome of a national policy, are likely to be indicative of what happened across the nation. They have important implications for the sector as a whole in terms of funding, decentralization, health facilities, sector performance, and the direction in which the sector is heading.

Clearly any increase in the number of staff also increases the salary bill, already running at 40% of all public expenditure for health at the district level in these districts [5]. Increasing the proportion of healthcare providers who are PNS has the effect of increasing the cost of salaries and benefits (including their own health care) for civil service staff, the item that, under Indonesia's decentralization, has first call on the general allocation fund from the central government. Thus, in the absence of a matching increase in the general allocation fund, those available for operations expenses are decreased by the extent to which salaries increase. Because the decision to increase the numbers of PNS and the use of central transfers to meet the additional salary costs are made by the central government, the funds over which the district has discretion are also decreased, an action that continues the central claw back of control over funds supposedly, under Indonesia's decentralization [6], now under the control of the district. At the same time, the districts have replaced many of the staff converted to PNS with additional staff on local contracts. Even though it was not possible to repeat the detailed assessment of use of public funds made for 2006 [5], we know that an increase in PNS numbers decreases the funds over which the district has control. At the same time this central decision relieves district authorities of the need to increase productivity and rationalize their staffing patterns and levels - why worry when the central government will continue to pay. Districts had been creating some flexibility in their hiring patterns through the use of contract staff. Although it appears that flexibility is now reduced as the central government has prohibited districts from hiring contract staff, in reality it seems that is not the case for despite this 'ban', districts are still hiring staff on contract, apparently without incurring sanctions from the central government. The overall effect is that the public sector salary bill for the health sector has increased.

The public portion of the Indonesian health system has low levels of productivity [P Heywood, NP Harahap. Health centre productivity in West Java Province, Indonesia. Unpublished] and the performance of the sector is inadequate [7]. In addition, the quality of care in Indonesia is low [8-10]. Merely increasing the total number of staff or the number who are permanent civil servants without addressing the more systemic issues [7] is unlikely to raise the quality or the overall performance of the system.

Finally, this increase in the overall number of providers in the system also results in an increase in the private sector facilities - across the 3 districts, the 680 additional staff are associated with 511 additional facilities, 369 solo-provider facilities and 142 additional multi-provider facilities. As is already the case, the quality of these additional services (public or private) is also likely to be limited and there is an urgent need for the public sector to take its stewardship functions seriously. Even so, the district governments have few resources devoted to oversight of the quality of care. An increase in the PNS staff alone without serious efforts to monitor service quality is unlikely to lift the mediocre performance of the sector.

Whilst this increase in overall staff levels (and, indirectly, facilities) and in the number and proportion of permanent civil servants might be applauded as an attempt to improve the low density of health service providers in Indonesia [11] its effect on health system performance is likely to be limited because it is not part of an overall coherent approach to improving the performance of the health sector [7]. At the same time the health information system is unable to provide the district level information needed to track changes and understand human resources for health at the district level. Because policy for human resources in health is weak and civil service reform has stalled, the central, provincial and district governments appear to be operating independently with respect to human resources in health. For political reasons the central government decided to convert various forms of contract workers (mostly PTT for doctors and midwives and only local contract for nurses) to PNS. The West Java Provincial government has been using its own resources to fund extra doctors and other health staff through a provincial contract scheme and BHL (a provincially-funded scheme which is found only in West Java). The districts have decided to maintain flexibility of hiring through the use of 'new' categories of local contracts. Each level of government responds to a different constituency and independently of the other. The human resources for health policy is uncoordinated and weak. Further, there is no overall health strategy which addresses the health problems of at least the next 30 years to provide a context for the development of policy about its most important asset, human resources. Indonesia needs both the strategy and the policy as soon as possible.

## Competing interests

The authors declare that they have no competing interests.

## Authors' contributions

PH and NPH conceived the study and drafted the manuscript. SA collected the data and assisted with interpretation of the results. All authors reviewed the final manuscript.

## Footnotes

^1 ^Doctor, nurse or midwife who works for a health facility on a local government contract. Paid, hired and fired by the district government from its own budget. Terms and conditions of their employment are not well documented and there is variation between facilities and districts.

^2 ^Health facility is defined as a physical structure (which varies from a large complex of buildings to a single room in a house) from which health services are offered by a doctor, nurse or midwife. See [2] for definitions of each facility type.
